# Ocular manifestations of monkeypox: correspondence

**DOI:** 10.5935/0004-2749.2022-0359

**Published:** 2023-04-10

**Authors:** Pathum Sookaromdee, Viroj Wiwanitkit

**Affiliations:** 1 Private Academic Consultant, Bangkok, Thailand; 2 Honorary professor, Dr DY Patil Vidhyapeeth, Pune, India

Dear Editor,

We would like to share ideas on the publication “Ocular manifestations of monkeypox: a case report^(1)^.” In this report, Nogueira Filho et al. described a patient with monkeypox who had ocular complaints (eye irritation and conjunctivitis) and identifiable conjunctival lesions on biomicroscopy and fluorescein tests^(1)^. According to the authors, the ophthalmological signs are still not well understood^(1)^. We believe that the key to controlling monkeypox is early diagnosis. The present monkeypox outbreak is difficult for doctors to control. Laboratory testing is typically used to determine the presence of monkeypox. However, clinicians must first make a provisional clinical diagnosis or identify a suspicious case before the laboratory analysis can have any effect. The alleged clinical diagnosis might make things less dangerous. Skin lesions, which are exceptional in any manner, are rare unless specific conditions are met. Thus, it is critical to realize that certain people, such as those who have neurological or digestive problems, only display particular symptoms^(2,3)^. Another crucial element is the quality control of laboratory diagnostics. It remains a problem because laboratory diagnosis for monkeypox could lead to inaccurate epidemiological data^(4)^. The current case has an ocular presentation. Surely, the issue has been raised and might be conveniently ignored^(5)^. However, other complications and comorbidities are significant issues. Other sexually transmitted diseases, such gonorrhea, may sometimes co-occur but go unrecognized^(6)^. The ocular manifestation may be a clinical issue related to monkeypox or other unnoticed concomitant medical conditions.
