# Supplementary material for: Ocular manifestations of monkeypox: correspondence
Source: Arq Bras Oftalmol. 2023 Apr 10;86(2):192–3. doi: 10.5935/0004-2749.2022-0359 (PMC11892472; doi:10.5935/0004-2749.2022-0359)
Supplement: Related texts

# Reply: Response: Ocular manifestations of monkeypox: correspondence

**Authors:** Pedro Antonio Nogueira Filho, Carolina dos Santos Lazari, Celso Francisco Hernandes Granato, Marina Akiko Rampazzo Del Valhe Shiroma, Aline Lopes dos Santos, Mauro Silveira de Queiroz Campos, Denise de Freitas

**Affiliations:** 1 PEnsE, Research, Teaching and Experimentation, Vison One, Department of Ophthalmology and Visual Sciences, Escola Paulista de Medina, Universidade Federal de São Paulo, São Paulo, SP, Brazil.; 2 Department of Ophthalmology and Visual Sciences, Escola Paulista de Medina, Universidade Federal de São Paulo, São Paulo, SP, Brazil.; 3 Fleury Diagnostic Medicine, São Paulo, SP, Brazil.; 4 Department of Medicine, Discipline of Infectology, Escola Paulista de Medina, Universidade Federal de São Paulo, São Paulo, SP, Brazil.

Dear Editor,

We are honored to have our work mentioned, and we thank Drs Sookaromdee and Wiwanitkit for their recognition and contact.

We understand that the monkeypox-related ocular manifestations have been reported and that monkeypox can manifest in the eyes. For example, we already have two cases under follow-up that evolved with manifestations linked to the corneas and conjunctiva and are being followed up for around 90-120 days^(1)^.

## References

[7] Abdelaal A, Serhan HA, Mahmoud MA, Rodriguez-Morales AJ, Sah R (2022). Ophthalmic manifestations of monkeypox virus. Eye (Lond).
